# Restricted duty hours for surgeons and impact on residents quality of life, education, and patient care: a literature review

**DOI:** 10.1186/1754-9493-3-3

**Published:** 2009-02-20

**Authors:** Hans-Christoph Pape, Roman Pfeifer

**Affiliations:** 1 Department of Orthopaedic Surgery, University of Pittsburgh Medical Center, Kaufmann Medical Building, Suite 1010, 3471 Fifth Avenue, Pittsburgh, PA 15213, USA

## Abstract

**Background:**

Work-hour limitations have been implemented by the Accreditation Council for Graduate Medical Education (ACGME) in July 2003 in order to minimize fatigue related medical adverse events. The effects of this regulation are still under intense debate. In this literature review, data of effects of limited work-hours on the quality of life, surgical education, and patient care was summarized, focusing on surgical subspecialities.

**Methods:**

Studies that assessed the effects of the work-hour regulation published following the implementation of ACGME guidelines (2003) were searched using PubMed database. The following search modules were selected: work-hours, 80-hour work week, quality of life, work satisfaction, surgical education, residency training, patient care, continuity of care. Publications were included if they were completed in the United States and covered the subject of our review. Manuscrips were analysed to identify authors, year of publication, type of study, number of participants, and the main outcomes.

**Review Findings:**

Twenty-one articles met the inclusion criteria. Studies demonstrate that the residents quality of life has improved. The effects on surgical education are still unclear due to inconsistency in studies. Furthermore, according to several objective studies there were no changes in mortality and morbidity following the implementation.

**Conclusion:**

Further studies are necessary addressing the effects of surgical education and studying the objective methods to assess the technical skill and procedural competence of surgeons. In addition, patient surveys analysing their satisfaction and concerns can contribute to recent discussion, as well.

## Background

Resident work-hour restrictions have been implemented nationwide under the guidelines established by the Accreditation Council for Graduate Medical Education (ACGME) in July 2003 [1]. The main goal of this regulation is to improve patient safety and quality of care due to reduction of fatigue related medical adverse events. Numerous investigations previously demonstrated an association between the reduction in cognitive and manual skills induced by fatigue or sleep deprivation. These were thought to be associated with a high risk of medical errors in various subspecialties [2-6]. On the other hand, surgical experience (high case load) is known to be significantly associated with decreased complication rates and medical errors [7-10]. One may therefore argue that the limitation of work-hours during the surgical/orthopaedic residencies may negatively impact the education and technical competence. The effects of this regulation on surgical education and quality of patient care continue to be debated. In this review, studies published following the implementation of the ACGME reform are summarized. Data on effects of limited work-hours on the quality of life, surgical education, and patient care is presented, focusing on residents in surgical subspecialties.

## Methods

### Literature Search

To identify the relevant publications, a Medline database search of articles published following the implementation of ACGME guidelines (July, 2003) through PubMed was performed. Relevant studies were retrieved using the following sequences of key words: *work-hours, 80-hour work week, quality of life, work satisfaction, surgical education, residency training, patient care, continuity of care*. Synonyms were used to find further relevant literature. In addition, we reviewed the references from the resulting publications to identify further potential articles to be included in our study.

### Selection of Relevant Manuscripts

Studies were included that assessed the effects of work-hour limitations on residents quality of life, surgical education, and the quality and continuity of patient care, respectively. Publications were selected if they covered at least one subject of our review and were completed in United States. Review articles, textbook chapters, poster presentations, and abstracts were exluded from this study. Studies were eligible if they were prospective, retrospective, or descriptive.

### Analysis of relevant Papers

A total of twenty-one articles satisfied the inclusion and exclusion criteria for this analysis. We reviewed and summarized the findings published in the studies. Variables of interest included authors, year of publication, type of study, number of participants, and the main outcomes.

## Quality of life

Resident work hour regulations have been implemented in the State of New York during the 1980s [11]. It was reported that a majority of surgical residents experienced improved quality of life as a result of these work hour limitations [12,13]. Prior the nationwide implementation of ACGME requirements on resident duty hours, a survey performed by Whang and colleagues [13] characterized the perceptions and desires of surgical residents on the work hour reform. In this study, 81% of the respondents reported that sleep deprivation and the abusive work environment have negatively affected their work. Moreover, the majority of surgical residents supported work hour limitations and believed that the new regulation would have positive effects on the quality of their personal life [13]. Following the implementation, numerous investigations confirmed the anticipated improvements of residents quality of life (Table 1) [14-20]. According to the surgical residents, the regulation has had a positive impact on the lifes outside of the hospital; namely more time for reading, rest, time spent with their families and responsibilities apart of work [17]. Improved moral and decreased fatigue were also reported and mentioned as positive aspects [14,16,17,20]. Interestingly, a different perception was found between junior versus senior residents with senior residents being less enthusiastic and more dissatisfied with the new regulation [14,16]. The authors assumed that the internalized culture of surgery and transfer of work from junior to senior residents following the regulation affected the opinion of senior resident surgeons. Although not specifically mentioned, it is assumable that the differences in the daily activities may play a role. While junior residents are mainly involved in floor work, writing discharge summaries, and general patient care, senior residents spend a higher amount of time in the operating room and are learning the surgical profession and the skills required.

**Table 1:** Summary of studies analysing the impacts on surgeon's personal life

**Personal Life**					
**Improved**		**No Change**		**Worsen**	

**Source**	**Outcome**	**Source**	**Outcome**	**Source**	**Outcome**

**Basu et al, 2004**[14] *A 68-item survey of plastic surgery residents (n = 12)*	Resident quality of life and morale had improved	**Gelfand et al, 2004**[21] *Pre-post survey of residents (n = 37) and faculty members (n = 27)*	No changes in emotional exhaustion, depersonalisation, and personal accomplishment, no significant changes in residents burnout	_	_

**Chung et al, 2004**[15] *Pre-post survey of surgical residents.*	Improvements in fatigue-related issues, more work satisfaction, improvement of lifestyle	_	_	_	_

**Kort et al, 2004**[16] *Survey of general surgery residents (n = 164)*	Increased personal time and decreased fatigue at work, more time for family, senior residents were less enthusiastic than junior residents	_	_	_	_

**Stamp et al, 2005**[17] *Pre-post survey of surgical residents (n = 28)*	More time for rest, time with family, and socializing	_	_	_	_

**Hutter et al, 2006**[18] *Survey of surgical residents (n = 58) and surgical attending physicians (N = 58), web based MBI survey(Burnout)*	Decreased burnout scores, less emotional exhaustion, improved quality of life, increased motivation to work	_	_	_	_

**Immerman et al, 2007**[19] *Survey of opinions and attitudes of orthopaedic residents (n = 976) and program directors (n = 85)*	There was an overall agreement that the quality of life had improved	_	_	_	_

**Schneider et al, 2007**[20] *Pre-post evaluation of operative case logs, standardized scores, residents perception survey*	Substantial improvements of residents satisfaction and quality of life	_	_	_	_

One publication reports emotional exhaustion and depersonalisation among most residents before and after introduction of work-hour reduction, whereas, important clinical activities such time spent in the operating room, clinic, and making rounds did not changes as well [21].

In summary, almost all investigations have reported improved quality of life and work-related satisfaction in surgical residents. However, most authors agree that the working conditions cannot be improved by reducing work-hours only. It is concluded that due to limited work time, the work load should be distributed to colleagues or auxiliary staff (physician extenders).

## Education

Surgery is a procedure – based specialty requiring the acquisition of technical skills and procedural competence. This experience depends on the variety of cases and case load during the residency. Several surveys have demonstrated improvements in theoretical surgical education with a significant increase of American Board of Surgery In-Training Examination (ABSITE) scores as a consequence of additional time for reading, more time for self preparation, and for conferences [12,14,19,20]. Surprisingly, other studies did not find changes in operative volume and number of cases performed either by senior or junior residents after reduction of work-hours (Table 2 and Table 3) [17,18,21-26]. These findings appear to demonstrate that fewer work-hours do not jeopardize the volume of procedures and with it the procedural experience of surgical residents. However, there may be issues in terms of the appropriate documentation of the procedures. Also, most studies do not differentiate between the difficulty of the procedure (removal of hardware versus open reduction and internal fixation). Also, one may wonder if the 80 hour work rule has been violated and has remained undetected.

**Table 2:** Summary of studies analysing the impacts on surgeon's education (Part 1)

**Education I**					
**Improved**		**No Change**		**Worsen**	

**Source**	**Outcome**	**Source**	**Outcome**	**Source**	**Outcome**

**Basu et al, 2004**[14] *A 68-item survey of plastic surgery residents (n = 12)*	More time for general reading, preparation for operative cases, and for presenta-tions and for conferences	**Gelfand et al, 2004**[21] *Pre-post survey of residents (n = 37) and faculty members (n = 27), and work-hour regist,*	No significant changes in operating room hours, clinic time, and duration of rounds	**Chung et al, 2004**[15] *Pre-post survey of surgical residents, determination of program components*	Fewer consultations seen, reduced conference attendance, and reduced operation per week.

**Immerman et al, 2007**[19] *Survey of opinions and attitudes of orthopaedic residents (n = 976) and program directors (n = 85)*	Especially junior residents perceived that the new regulation has a positive effect on surgical education	**Ferguson et al, 2005**[24] *Pre-post study analysing surgical operative logs of residents in general surgery, restrospective review*	The residents operative volume could be maintained, the operative volume was unchanged	**Jarman et al, 2004**[27] *Pre-post study of general surgery residents, review of operative logs*	Work-hour restrictions result in a significant decrease in operative experience

**Schneider et al, 2007**[20] *Pre-post evaluation of operative case logs, standardized scores, residents perception survey*	Substantial increase of operative cases in PGY1 and PGY2, ABSITE Score improved	**Malangoni et al, 2005**[25] *Pre-post study of work-load and work-hours, senior residents survey at level I trauma center*	The number of operation performed by senior residents did not changed, no difference in trauma patient care exposure or operative case load	**Kort et al, 2004**[16] *Survey of general surgery residents (n = 164)*	The majority felt that their operative experience was reduced

_	_	**Mendoza and Britt, 2005**[23] *Mixed-design study of 253 programs, survey of residents, determination of operative volume*	No significant differences in the operative volume of residents	**Cohen-Gadol et al, 2005**[29] *Nationwide survey of program directors (n = 93) and residents (n = 617)*	61% of residents noted that the new guidelines have had negative effect on their training

_	_	**Spencer and Teitelbaum, 2005**[22] *Pre-post study and survey of residents (n = 91) on pediatric surgery*	No significant changes in total number of cases per day for junior and senior residents, residents perception of their education did not changed	**Myers et al, 2006**[28] *Pre-post survey of general surgery residents (n = 200) at 5 academic medical centers*	Decrease in the evailable opportunities for bedside learning. The quality of education may have declined

**Table 3:** Summary of studies analysing the impacts on surgeon's education (Part 2)

**Education II**					
**Improved**		**No Change**		**Worsen**	

**Source**	**Outcome**	**Source**	**Outcome**	**Source**	**Outcome**

_	_	**Stamp et al, 2005**[17] *Pre-post survey of surgical residents (n = 28)*	Residents felt that their training has not been affected significantly	**Peabody, 2006**[30] *Survey of orthopaedic program directors (n = 94) and senior residents (N = 59)*	The respondents thought that the regulation had a negative impact on orthopaedic residency education

_	_	**Hutter et al, 2006**[18] *Survey of surgical residents (n = 58) and surgical attending physicians (N = 58), ACGME case logs, ABSITE Score*	Resident training and education objectively were not statisticaly diminisched (ACGME case logs, and ABSITE Score)	_	_

_	_	**Pappas and Taegue 2007**[26] *Pre-post evaluation of case-log database. Single university based orthopaedic residency program*	The new regulation has not decreased the experience of orthopaedic residents	_	_

Many clinicians argue that such work hour limitations adversely affect the training experience and report reduced quantity of operations, reduced consultations seen, and reduced conference attendance [15,27,28]. This opinion was also reflected in surveys of surgical residents [16,29,30]. Kort et al [16] surveyed the resident perceptions of the impact of work-hour restrictions. In this study, the majority (57.3%) of the residents felt that restricted work-hours reduced their operative experience due to a reduction in the case load. Especially senior residents negatively judge the new work-hour regulation and expect adverse effects on surgical education [16].

In summary, it is still controversial whether the restricted work hours negatively influence the surgical education and training. The new regulation reduces the duration of stay in the hospital and may lead to fewer opportunities to participate in operative cases. Thus, less experience may ensue for the surgeon in training. Further observations and objective evaluations of the surgical education can indicate whether the ACGME work-hour policy has adversely affected the clinical experience. Certainly, the estimation of ABSITE scores is an objective method to analyze the theoretical education of surgical residents. Unfortunately, the degree of surgical experience, procedural competence, and technical skills are difficult to assess. Therefore, it has been tried to use analyses of surgical adverse events, mortality, and morbidity to assess operative skills [8,31-33].

## Patient care

The ACGME work-hour regulation caused the development of shift models with multiple residents being responsible for a given patient [34]. Thus, it has been discussed that the continuity of patient care may be compromized due to the lack of familiarity with patients. Numerous surveys studying resident perceptions performed prior and after the implementation of the work-hour reform have demonstrated that surgical residents and program directors expect adverse effects on the quality and continuity of patient care (Table 4) [13,15,16,28,29]. In this context, previous investigations found that the reduction of work-hours (from 12 to 8 h) in the ICU resulted in an increased frequency of complications and readmissions [35]. Other investigators report an association of cross-coverage in patient care with complications of medical therapy and preventable adverse events [36]. However, there is a lack of studies that demostrate advese effects in surgical patients after the new work-hour regulation. In addition to the analysis of surveys, several authors used objective methods (Mortality, Morbidity, Complication rates, and Missed Injuries) to evaluate the impact on quality of patient care [18,20,37-40]. In an recent study, Morrison et al [39] found a slight decrease of mortality and morbidity amoung trauma patients in university hospitals. Previous investigations have demonstrated that the mortality and morbidity most likely did not change following the implementation of the new work regulation [18,37,38]. Studies performed in the ICU support the results mentioned above and demonstrate a lower rate of serious medical errors and decreased attentional failures due to elimination of extended work shifts [41,42].

**Table 4:** Summary of studies analysing the impacts on quality of patient care

**Patient care**					
**Improved**		**No Change**		**Worsen**	

**Source**	**Outcome**	**Source**	**Outcome**	**Source**	**Outcome**

**Basu et al, 2004**[14] *A 68-item survey of plastic surgery residents (n = 12)*	Residents noted an improved ability to deliver patient care, hight consensus that this policy is benefitial for patient care	**Rogers et al, 2005**[40] *Pre-post study of complications, missed injuries, delayed diagnoses, and admission rates*	No significant difference in the overall complication rate, delayed diagnoses, or missed injuries	**Chung et al, 2004**[15] *Pre-post survey of surgical resident*	The new regulation reduced continuity of care, reduced consultations seen

_	_	**Hutter et al, 2006**[18] *Prospective study of mortality, complication rates, NSQIP*	No significan difference in quality of patient care, no differences in mortality rates	**Kort et al, 2004**[16] *Survey of general surgery residents (n = 164)*	Continuity and safety of care were perceived negatively by surgical residents

_	_	**Schneider et al, 2007** *Pre-post evaluation of operative case logs, standardized scores, residents perception survey*	Patient outcome measures, including monthly mortality and number of admissions showed no changes	**Cohen-Gadol et al, 2005**[29] *Nationwide survey of program directors (n = 93) and residents (n = 617)*	93% thought that the new reform has had a negative impact on continuity of patient care.

_	_	**Shetty et al, 2007**[37] *Pre-post study of mortality rates in 551 community hospitals (n = 1.511.945 patients)*	In surgical patients there were no significant changes of mortality rates	**Myers et al, 2006**[28] *Pre-post survey of general surgery residents (n = 200) at 5 academic medical centers*	Errors related to reduced continuity of care significantly increased. The continuity of care had decreased a lot.

_	_	**Volpp et al, 2007**[38] *Pre-post study of mortality rates in 3321 nonfederal hospitals (n = 8.529.595 patients)*	The new reform was not associated with either significant worsening or improvements of mortality	_	_

_	_	**Morrison et al, 2008**[39] *Retrospective pre-post study of mortality, length of hospitalisation Data from NTDB*	This analysis demonstrates slightly decreased mortality and morbidity rates. But more likely clinically not important	_	_

According to several surveys, surgical residents are concerned about the negative impacts on patient continuity and quality of care. Otherwise, objective studies have demonstrated that the quality of care (mortality and morbidity rates) of surgical patients rather did not change following the implementation of the ACGME work-hour reform. Certainly, particularly the patient-resident relationship and previous patient focused attitude in treatment may suffer under the new regulation. Whether these changes also have an impact on patient satisfaction has yet to be determined. Surveys among patients are missing to date.

## Strategies to comply with the ACGME work-hour regulation

To comply with the guidelines of the work-hour regulation, residency programs have to reduce or distribute the work-load that would have been completed by residents. Several benefits were reported after the incorporation of physician extenders (nurse practitioners or physician assistants) into the surgical service [43-45]. Studies published following the implementation of the ACGME work-hour reform support prior experience [46-52]. These showed a significant decrease of work-hours, workload, and even increased resident time in the operating room due to reduction of noneducative and nonclinical tasks after physician extenders were employed [46,47]. Moreover, residents were satisfied with this assistance and believed that it decreased stress levels and helped to improve morale [47,51]. These results demonstrate that physician extenders could be successfully integrate into surgical service and effectively improved resident work outlook [46,47,49,51]. According to the studies, hiring of physician extenders was the most commonly used strategy to comply with ACGME work-hour reform [48,50]. To maintain the continuity of patient care, authors introduced and evaluated a night-float system, which appears to reduce resident fatigue, increase sleep efficiency, and maintain quality and continuity of patient care as judged by patients and nurse [34,50].

## Conclusion

Our review appears to support the following main findings: The new work-hour regulation improves the quality of life and work-related satisfaction especially in junior residents. In contrast, senior residents were less satisfied with the new reform. The effects on surgical education remain unclear due to inconsistency in studies, problems in assessment of the quality of the education and the quality of the surgeon's performance. Also, there appears to be no evidence whether the reduction of work-hours and the expected decrease of fatigue increased patient safety. Moreover, objective studies demonstrate no changes in mortality and morbidity following the implementation, although, residents perceptions regarding the quality of patient care were mostly negative. Finally, we observed a lack of patient surveys analysing patient satisfaction, quality and changes of care. This data could contribute to the recents debate. Moreover, further studies are necessary to analyse the effects on scientific research in teaching hospitals, to evaluate the changes of medical error and complication, and to assess the development of costs.

## Competing interests

The authors declare that they have no competing interests.

## Authors' contributions

All authors were involved in the research project and preparation of the manuscript. PHC: He made a substantial contribution to conception and design, and gave a critical and final approval. PR: He has collected the data and made an analysis and interpretation of these data. He also made a draft of the manuscript and revisions. All authors read and approved the final version of the manuscript.
